# Soil transmitted Helminthiasis and associated risk factors among elementary school children in ambo town, western Ethiopia

**DOI:** 10.1186/s12889-017-4809-3

**Published:** 2017-10-10

**Authors:** Fikreslasie Samuel, Asalif Demsew, Yonas Alem, Yonas Hailesilassie

**Affiliations:** Ambo University, Ambo, Ethiopia

**Keywords:** Soil transmitted Helminthiasis, Risk factors, School children, Ethiopia

## Abstract

**Background:**

Soil-transmitted helminths (STHs) are widespread in underdeveloped countries. In Ethiopia, the prevalence and distribution of helminth infection varies by different exposing risk factors. We therefore investigated the prevalence of and risk factors of STHs infection in school children living in Ambo town, west Shoa Ethiopia.

**Methods:**

In 2014/15, among 375 school children planed to be included in this study, only 321 school children were recruited in the study. Data onto school children from different schools were collected, including stool samples for qualitative STHs analysis. Questionnaire data on various demographic, housing and lifestyle variables were also available.

**Results:**

Prevalence of any STHs infection was 12.6%. The respective prevalence of major soil-transmitted helminths is *Ascaris* (7.8%), Hookworm (2.8%) and *Trichuris* (2.2%). This study result shows STHs prevalence varies regards to age, sex, latrine use, family size and nail trimming.

**Conclusion:**

The results of the present study indicated that the percentage of positive finding for STHs in Ambo area is low. Besides, Large Family size, not nail trimming and unavailability of improved latrine were identified as predisposing factor for STHs infections. All school children enrolled and not enrolled in this study should be treated twice a year until the prevalence falls below the level of public health importance.

## Background

Soil-transmitted helminthiases are a group of parasitic diseases caused by nematode worms that are transmitted to humans by faecally contaminated soil. These are among the most prevalent infections of humans living in sub Saharan Africa countries. The latest estimates indicate that more than 2 billion people are infected with these parasites [1, 2]. The highest prevalence occurs to areas where sanitation is inadequate and water supplies are unsafe [1].

The soil-transmitted helminths (STHs) of major concern to humans are *Ascaris lumbricoides*, *Trichuris trichiura*, *Necator americanus* and *Ancylostoma duodenale*. According to reports in 2010, an estimated 819.0 million people were infected with *A. lumbricoides*, 438.9 million with Hookworm and 464.6 million with *T. trichiura.* [3]. STHs light infections usually have no symptoms. However, heavier infections cause a variety of symptoms including malnutrition, malabsorbition, abdominal pain, cramping and tiredness, and impaired cognitive and physical development. The worms feed on host tissues, including blood, which leads to a loss of iron and protein. The worms also increase malabsorption of nutrients. In addition, some soil transmitted helminths cause loss of appetite and, therefore, a reduction of nutritional intake and physical fitness. The nutritional impairment caused by soil transmitted helminths is recognized to have a significant impact on growth and physical development [4, 5].

Soil transmitted helminths infection are distributed over the world with high prevalence rates in tropical and sub-tropical countries those with lack of adequate sanitary facilities, inappropriate waste disposal systems, lack of safe water supply, and low socio- economic status. School age children mainly are at high risk of these intestinal parasitic infections especially in developing countries like Ethiopia [1]. Though there are previous data on prevalence and associated risk factors of soil transmitted helminths infection among these high risk groups in different areas of Ethiopia, it is unknown in this study area. Hence, this study was aimed to assess the prevalence and associated risk factors of STHs among elementary school children from December 2014 to April, 2015 at Ambo town, Western Ethiopia.

## Methods

### Study area and population

The study was conducted in Ambo town, Western Ethiopia, from October, 2014 to May, 2015. Ambo is located 115 km from Addis Ababa in West Shewa Zone, Oromia Region. It situated at latitude and longitude of 8°59′N 37°51′E and an elevation ranges from 1900 to 2275 m above sea level. The population size was 76,774, of whom 39,155 are males and 37,619 are females and are ethnically mixed. It has an annual rainfall and temperature ranging from 800 to 1000 mm and 20-29 °C respectively [6]. Agriculture is the main occupation of the population of the area. The agricultural activities are mainly mixed type with cattle rearing and crop production under taken side by side [7]. There are nine government and eleven private elementary schools in the town. Besides, one governmental hospital, two health centers and 20 private clinics are found in the town, serving the community to improve the health problem.

### Study design and sample size determination

A cross sectional survey was conducted from December 2014 to April, 2015 among primary school children. The sample size for this study was calculated by using single proportion formula at 95% confidence interval (CI) level (Z (1-ά/2) = 1.96), with prevalence of 66.7% from previous similar study conducted in North Gondar in 2014 [8] and 5% marginal error. Then the sample size was calculated as n = [Z 1- a/2] 2 P (1-p]/d2, Where: n = sample size, P = prevalence of parasites from previous studies, Z 1-a/2 = CI of 95%, d = Marginal error. By adding 10% of contingency, 375, but only 321 study subjects were participated in the study due to many reasons.

**Fig. 1:**
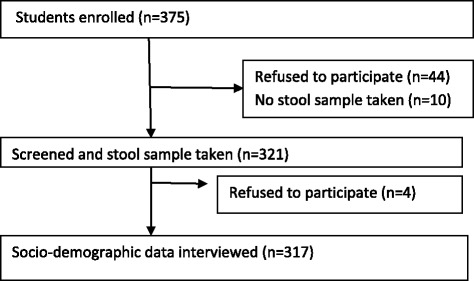
Trial diagram: soil transmitted helminthiasis and associated risk factors among elementary school children in ambo town, western Ethiopia, 2015

To determine the proportion of students participate in the study, schools were selected with simple random sampling technique. Similarly, from selected schools grades and sections were selected by lottery method. Finally, students (subjects) from each section were selected with systematic random sampling proportionally by dividing the total sample size to the sections by using their registration book as sampling frame. Children attending school in the selected elementary schools during the study period, who were voluntary to participate in the study and able to provide stool sample within the study period were included in this study, i.e., Liben mecha (*n* = 140); Oddo liben (*n* = 35); Senkele (*n* = 48); Feature generation (*n* = 43); Adventist (*n* = 16); Kidanmihret (*n* = 39). Brief description of students enrolled and not enrolled in the study is expressed in Fig. 1.

### Demographic factors assessment

Data on socio demographic factors and other potential risk factors for STHs infection related to age, family size, latrine use and others were collected using pretested questionnaire prepared for the purpose of this study. The questionnaire was prepared in English and then translated to Oromiffa and checked for fitness. Trained Health extension workers who were fluent with local languages (Amharic and Oromifa) interviewed study participants. Demographic factors were assessed to determine the potential of these factors to expose for STHs infection.

### Stool sample collection and examination

Sufficient amount of stool specimen (approximately 5 g) was collected from each participant using a leak proof, tightly corked plastic container. The stool samples were labeled processed and examined using formol-ether concentration method at Ambo Hospital according to WHO methods [9]. Microscopic examination was performed with 100X and 400X magnifications. The result was recorded carefully on well prepared format for this purpose. The samples were examined within the 30 min of preparation of the sample. The intensity of infection was estimated through referring the cut off values for classes of intensity of STHs infections, after determining the eggs per gram of stools. For quality control reason, from all of the slides, 10% was randomly selected and re-examined at the end by experienced laboratory technologist who was blinded for the first examination results.

### Data analysis

Data was entered and analyzed using SPSS version 20.0 computer software after checking its completeness. The dependent variables were any STHs infection in the school children (where STHs infection is taken to mean infection with any of *A. lumbricoides*, *T. trichiura*, Hookworm or others). Potential risk factors explored were demographic factors (sex, age, family size and latrine use), markers of social advantage and factors associated with housing and lifestyle. It was summarized in percentages and presented in tables. Pearson’s Chi-square test was performed where appropriate to identify any association between STH infection and independent factors. Association between risk factors and parasitological test results was assessed. *P*-value less than 0.05 were considered as statistically significant.

### Ethical considerations

This study was reviewed and ethically approved by the Ethics Review Committee on Health Research, Faculty of medicine and health sciences, Ambo University. Permission to conduct the study was also obtained from Ambo town Health Office, Educational Bureau, and School Principals.

The objective of the study was explained to school teachers and students at the time of baseline data collection. The stool sample was collected after obtaining written consent from parents/guardians and assent from children participated in the study. Positive individuals for STHs, *H. nana* and *S. mansoni* infections were treated with the standard dose of albendazole and praziquantel. The single-dose treatment of albendazole and praziquantel were administered under the supervision of medical doctor.

## Results

### Prevalence of soil-transmitted helminths

**Fig. 2:**
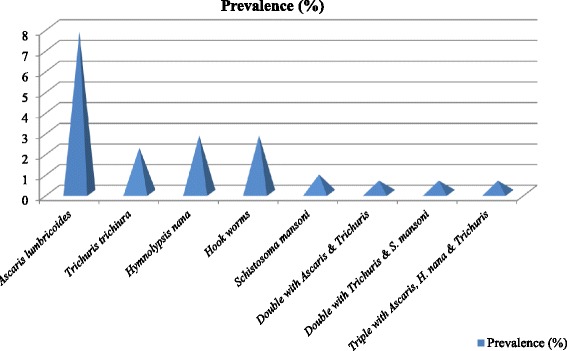
Prevalence of intestinal helminths among school children in Ambo town, western Ethiopia, 2015

Out of 321 stool specimens’ collected and examined using formol-ether concentration method, 59 (18.4%) were found positive for one or more helminth infections. Three species of STHs were identified in the stool samples, with the overall prevalence of any STHs infection being 12.8% in school children. *A. lumbricoides* was the predominant intestinal helminth infection, detected in 25(7.8%) of school children and Hookworm was the second most frequently detected intestinal parasite 9(2.8%) and the least predominate STH infection was *T. trichiura,* detected in 7 (2.2%) of school children. Moreover, intestinal parasites namely; *H. nana* and *S. mansoni* were also detected in 9(2.8%) and 3(0.9%) of school children, respectively as shown in Fig. 2.

Regarding to double infection, *A. lumbricoides* and *T. trichiura* as well as *T. trichiura* and *S. mansoni* were found similarly in 2(0.62%) of children. Besides, triple infection of *A. lumbricoides*, *H. nana* and *T. trichiura* were found in 2 (0.62%) of the kids.

The prevalence of intestinal helminths varies among schools as shown in Table 1. *Trichuris* infection was found to be prevalent only in two schools namely: Oddo liben and Liben mecha, respectively. Whereas, *Ascaris* infection was found among four primary schools: Liben mecha, Oddo liben, Senkele and Feature generation and predominated in Oddo liben primary school and slightly occurred in remaining above mentioned schools. Besides, Hookworm was prevalent in Senkele primary school than others, about 14.6% of the case was found in this school.

**Table 1:** Prevalence of intestinal helminths infection among primary schools in Ambo town, 2015

Type of parasitic infections	Helminths infection among schools					
	Liben mecha No (%)	Oddo liben No (%)	Senkele No (%)	Feature generation No (%)	Kidanemihret No (%)	Adventist No (%)
*Trichuris trichiura*	4 (2.86%)	3 (8.57%)	0 (.0%)	0 (.0%)	0 (.0%)	0 (.0%)
*Hymenolepis nana*	8 (5.71%)	0 (.0%)	0 (.0%)	0 (.0%)	1 (2.56%)	0 (.0%)
*Ascaris lumbricoids*	11 (7.86%)	10 (28.6%)	1(2.1%)	3 (6.98%)	0 (.0%)	0 (.0%)
Hookworms	1 (0.71%)	1 (2.86%)	7 (14.6%)	0 (.0%)	0 (.0%)	0 (.0%)
*Schistosoma mansoni*	2 (1.43%)	0 (.0%)	0 (.0%)	0 (.0%)	1 (2.56%)	0 (.0%)
Double infection with *Ascaris* & *Trichuris*	0 (.0%)	2 (5.71%)	0 (.0%)	0 (.0%)	0 (.0%)	0 (.0%)
Double infection with *Trichuris* & *S.mansoni*	1 (0.71%)	0 (.0%)	0 (.0%)	0 (.0%)	1 (2.56%)	0 (.0%)
Triple with *Ascaris*, *H. nana* & *Trichuris*	1 (0.71%)	1 (2.86%)	0 (.0%)	0 (.0%)	0 (.0%)	0 (.0%)

No. = number of positive cases

In this study proportions of classes of intensity of infections for the major STHs among school children were computed. The proportions of light infections for Hookworm, *A. lumbricoides* and *T. trichiura* were 100% (9), 100% (25), and 100% (7), respectively. Heavy and moderate infections were not observed in all helminths infection. The intensity of infection for all detected intestinal parasites was light.

### Socio-demography characteristics and risk factors for soil-transmitted helminthiasis

In this study, the analysis showed that the prevalence of STHs infection was not significantly associated with age of children. However, the prevalence STHs among 11–15 age group children was slightly higher than 6–10 age groups. STHs prevalence is significantly different with sex of the children. Regard of the family size, when the family number increases the occurrence of STHs infection was also increased. Family size is significantly associated with prevalence of STHs infection. Kids without latrine in their home were more affected than who have the latrine. As well, those who have traditional pit latrine are more exposed with intestinal helminths than have ventilated and improved latrine. However, those with water flash were not affected. See Table 2 below.

**Table 2:** Association between demographic factors and the prevalence of STHs in Ambo town, western Ethiopia, 2015

Socio-demographic factors		STHs infection status		Total	X^2^	P-value
		Negative	Positive			
Age	6–10	156 (81.7%)	35 (18.3%)	191 (100.0%)	0.49	0.12
	11–15	102 (81.0%)	24 (19.0%)	126 (100.0%)		
Gender	Girl	145 (78.4%)	40 (21.6%)	185 (100.0%)	0.068	0.031
	Boy	113 (85.6%)	19 (14.4%)	132 (100.0%)		
Family size	1 parent and child	4 (80.0%)	1 (20.0%)	5 (100.0%)	0.03	0.0
	1 parent and 2 children	15 (88.2%)	2 (11.8%)	17 (100.0%)		
	2 parents and 2 children	40 (88.9%)	5 (11.1%)	45 (100.0%)		
	2 parents & > 2 children	193 (79.4%)	50 (20.6%)	243 (100.0%)		
Latrine available	Yes	216((83.7%)	42 (16.3%)	258 (100.0%)	0.09	0.05
	No	36 (67.9%)	17 (32.1%)	53 (100.0%)		
Type of latrine	Ventilated improved	50 (92.6%)	4 (7.4%)	54 (100.0%)	0.036	–
	Traditional pit latrine	152 (79.6%)	39 (20.4%)	191 (100.0%)		
	Water flash	13 (100.0%)	0(.0%)	13 (100.0%)		
Finger nail trimming	Yes	115 (85.8%)	19 (14.2%)	134 (100.0%)	.039	.024
	No	31 (72.1%)	12 (27.9%)	43 (100.0%)		

## Discussion

The formol-ether concentration technique is widely used technique to recover helminths eggs. It is performed in order to separate the parasites from fecal debris. Such techniques not only increase the number of parasites in the sediment but also unmask them, making them more visible by removing organic and inorganic debris [10]. In this study, all samples were processed and examined using formol-ether concentration method for better results.

The overall prevalence of helminths infection found in the present study (18.4%) was relatively lower than reported from other parts of Ethiopia by [11–14]. Besides, Afework Bitew A, et al. [15] was also reported higher overall prevalence (65.6%) of helminth infection than in the present study. Factors like: Personal hygiene, water supply, latrine type, socioeconomic status of the community, living status, heterogeneity and educational status, contribute to the differences in the prevalence and distribution of these intestinal helminths.

The prevalence of STHs infection (12.4%) in current study was much lower than the prevalence among school aged children conducted in various areas of Ethiopia [2, 14, 16–18]; in Kenya (22% to 71%) [19]; in Honduras (72.5%) [20]; in China [21]. In contrast, the prevalence of STHs in Babile town, eastern Ethiopia (0.47%) [22], is relatively lower than the current result found in Ambo town, western Ethiopia. Such variation among these different communities might be due to several factors, which may affect transmission of STH infections. These factors could be: population genetic variation, age, multiparasitism, study time, technique used to detect the parasite, sanitation, type of weather, and altitude [23, 24].

In the present study, *A. lumbricoides* (7.8%) was found to be the pre dominant STHs, this is related with many studies [2, 18, 25–27], followed by Hookworm (2.8%) *and T. trichiura* (2.2%). The prevalence of Hookworm infection was comparatively lower than the prevalence of Hookworm infection previously reported from other regions of Ethiopia [13, 16], and also lower than the prevalence reported from Langano area [11]. The prevalence of *A. lumbricoides* and *T. trichiura* infections observed in the present study was 7.8% and 2.2%, respectively. These were higher than the prevalence reported from the study conducted in Ethiopia [28], but much lower than that reported from Wondo Genet, Southern Ethiopia [16, 29]. Such variations in prevalence of helminth infections are attributable to several risk factors, including low standard of living, poor socioeconomic status, poor personal hygiene and environmental sanitation, urbanization, human behavior, household clustering, occupation and climate. Moreover, Absence of public health education, deworming history, source of drinking water as well as absence of latrine could also contribute for different positive cases [30].

Regarding to the prevalence of STHs infection per school, *Ascaris* was the first predominated infection (28.6%), which was found in Odo liben primary school. Whereas, Hookworm was detected as second predominant infection (14.6%) among schools, found in Senkele primary school, which is the nearest primary school to rural areas. The third predominant parasite was *Trichuris* (8.57%) prevalence rate found in Oddo liben primary school. Over all, the prevalence of STHs was higher in nearby schools to rural areas than schools found in center of the town. Possibly, almost all of the school students were came from rural areas where shoe wearing practice and sanitation facilities are absent, which agreed with several studies conducted in the same country [31, 32].

The prevalence was varying as a result of many pre disposing factors. In this study, the analysis showed that the prevalence of STHs infection was not significantly associated with age of children found between 6 and 15 age groups. This outcome is concurred with previous studies [30, 32, 33]. However, the disease prevalence varies among girl and boy students and significantly different, which differed from the study conducted by Seid et al. [30]. Whereas, girl students were more affected than boy students, which is in agreement with the study by Mekonnen et al. [33]. Regard of the family size, when the family number increases the occurrence of STHs infection was also increased. Family size is significantly associated with prevalence of STHs infection.

Besides, the prevalence in this study was varying significantly with the type of latrine used. In this study, Kids without latrine in their home were more affected than who have the latrine. Similarly, Abossie and Seid found the same evidence in Chencha town, Southern Ethiopia [25]. But, contrast results were also reported [12]. As well, those who have traditional pit latrine are more exposed with intestinal helminths than have ventilated and improved latrines. However, those with water flash were not affected. Low improved latrine coverage, inadequate health education about sanitation and absence of maintenance of latrines could contribute for these differences. These are the ideal indication of the importance of improved latrine to control STHs [34]. Furthermore, kids without the habit of nail trimming were more exposed for STHs infection than who had. The following studies supported this outcome [14, 31, 35, 36].

## Conclusions

The results of the present study indicated that the percentage of positive finding for STHs in Ambo area is low. Besides, Large Family size, not nail trimming and unavailability of improved latrine were identified as predisposing factor for STHs infections. All school children enrolled and not enrolled in this study should be treated twice a year until the prevalence falls below the level of public health importance. Access to improved latrine could also improve for reduction of STHs prevalence below the percentage found in this study.

## Abbreviations

CI: Confidence interval
No.: Number of positive cases
SPSS: Statistical Package for Social Science
STHs: Soil-transmitted helminths
WHO: World Health Organization
